# A Bibliometric Analysis of Publications on Spinal Cord Injury Treatment With Glucocorticoids Using VOSviewer

**DOI:** 10.3389/fpubh.2022.907372

**Published:** 2022-08-08

**Authors:** Yu Zhang, An-An Li, Shi-Ning Xiao, Nan-Shan Zhong, Wei-Lai Tong, Shi-Jiang Wang, Jia-Ming Liu, Zhi-Li Liu

**Affiliations:** ^1^Medical Innovation Center, The First Affiliated Hospital of Nanchang University, Nanchang, China; ^2^Institute of Spine and Spinal Cord, Nanchang University, Nanchang, China

**Keywords:** bibliometric analysis, spinal cord injury, methylprednisolone, glucocorticoid, Web of Science

## Abstract

**Background:**

Spinal cord injury (SCI) has devastating physical and social consequences for patients. Systemic administration of methylprednisolone (MP) at a higher dosage though can reduce neurological deficits following acute SCI. Still, this treatment regimen is controversial, owing to the apparent dose-related side effects and relatively minor improvement in neurological function. Therefore, this study aimed at the bibliometric analysis of published literature related to SCI treatment, which may lead to future research trends.

**Methods:**

The literature published relating to SCI and using glucocorticoids for its treatment between 1982 and 2022 was collected and scanned in the Web of Science collection database using the keywords glucocorticoid, dexamethasone, MP, corticosteroids, and SCI, followed by using VOSviewer for bibliometric analysis of these articles.

**Results:**

A total of 1,848 published articles and 7,448 authors on SCI and glucocorticoid usage were identified. The SCI total link strength accounts for 1,341, and MP for 762 has a strong link to neuroprotection and inflammation. The mean citation count for the top 20 most-cited articles was 682 (range: 358–1,828), where most of these were descriptive studies having focused on clinical features. The Journal of Neurotrauma was the highest-ranked journal with 6,010 citations. A total of 69 articles were published by Michael G Fehlings from the University of Toronto with 6,092 citations. The University of Toronto has published 90-related manuscripts with 7,632 citations. In contrast, 800 articles were published in the United States, with 39,633 citations and total link strength of 5,714. The second-ranked country was China, with 241 published articles and 3,403 citations.

**Conclusions:**

The research published on applying MP in treating SCI has increased with time. Although the United States has made a significant global contribution to this important field of research, it requires rigorous clinical trials designed to verify the therapeutic role of MP in SCI and its appropriate dosage to find solutions for neurological recovery.

## Introduction

Spinal cord injury (SCI) can lead to sensory and motor nerve dysfunction and even autonomic loss in patients, drastically affecting the physical and social characteristics of patients with an added financial burden to health systems (1). As many as half a million people damage their spinal cord each year (2). For almost all of them, the injury will be life-changing. Acquired SCI has a variety of causes (3), such as traumatic events, inflammatory, infective, and neoplastic. The most common and severe injuries to SCI are traumatic in nature. Traumatic injuries lead to variable degrees of permanent neurological dysfunction. Because the poor prognosis of SCI is yet not addressed, the disability rate for this disease is extremely high (4).

The bibliometric analysis is a statistical method with the ability of quantitative analysis of published research papers related to a specific topic employing various mathematical models (5), enabling the analysis of thousands of published articles related to a defined research field and could help reveal top prominent publications and authors, collaborative linkages between authors and active journals (1). Furthermore, it helps access quality studies, identifies key research areas, and may help to predict future research directions (6). The Web of Science (WOS) online database is one of the most reputable citation databases, which includes almost all significant research papers and has a great influence and authority worldwide (6). In this study, WOS search results were exported to VOSviewer software for further analysis to derive research directions and priorities in selected areas (7).

To date, no bibliometric analysis of glucocorticoids usage in SCI has been reported yet. Due to the unavailability of an appropriate treatment protocol for SCI (8), the disability rate remains high (9). MP pulse therapy is a definitive treatment for SCI (10), generating diverse opinions among various authors. Hence, more knowledge could be extracted from published research (11). Our study takes the novel perspective of bibliometrics and attempts to understand the pros and cons of current treatment options and an exploration of future research directions.

## Methods

The global articles about SCI and glucocorticoids between 1982 and 2022 were searched in WOS from database inception to February 20, 2022. The search terms were presented as follows: glucocorticoid, dexamethasone, MP, corticosteroids, and SCI.

The information extracted met the preset requirements, which comprised basic information about literature such as counts of citation, title, year of publication, and journal. This information was exported into several text formats. All data were retrieved on the 20th of February 2022. The data were exported into a VOSviewer (version 1.6.18 Leiden University, Leiden, Netherlands) software for further analysis, which could generate map and cluster visualization (6, 12).

## Results

### Bibliometric Analysis of Annual Publication Output From 1982 to 2022

**Figure 1:**
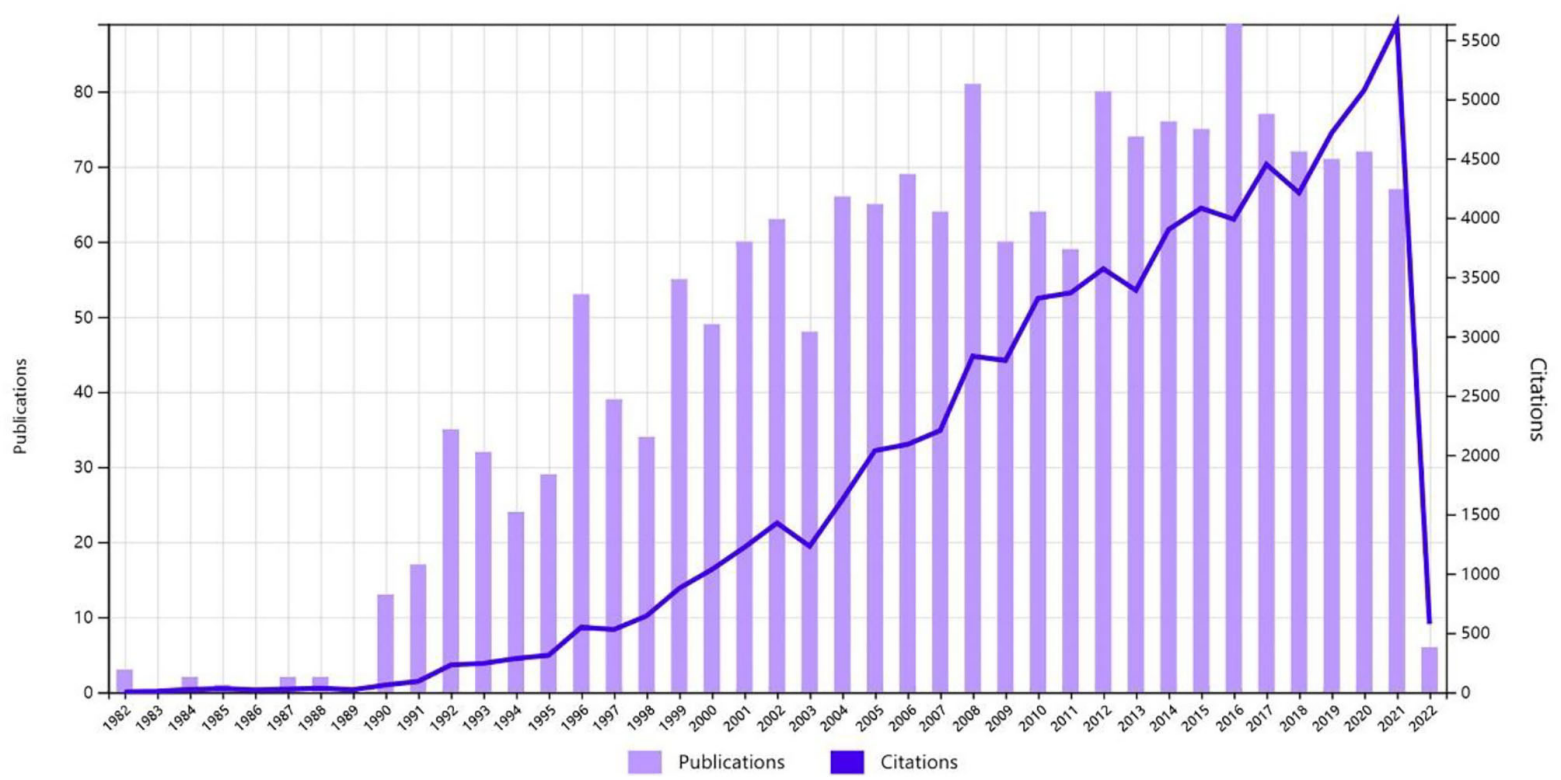
The distribution depicts years of publication, and the number of citations received.

About 1,848 publications about SCI and glucocorticoids were identified in the WOS database between 1982 and 2022, such as 1,477 (79.92%) original research articles, 227 (12.28%) review articles, and 144 other forms of publications, such as editorials, proceedings, etc. Among them, 1,324 (75.7%) papers were published before December 30, 2015, followed by an increasing trend of 70 published papers/year in the WOS core database, where the highest number of studies was published in 2016 (89 papers). The distribution depicting years of publication and the number of citations received are presented in Figure 1.

### Analysis of the Keywords and Visualization

**Figure 2:**
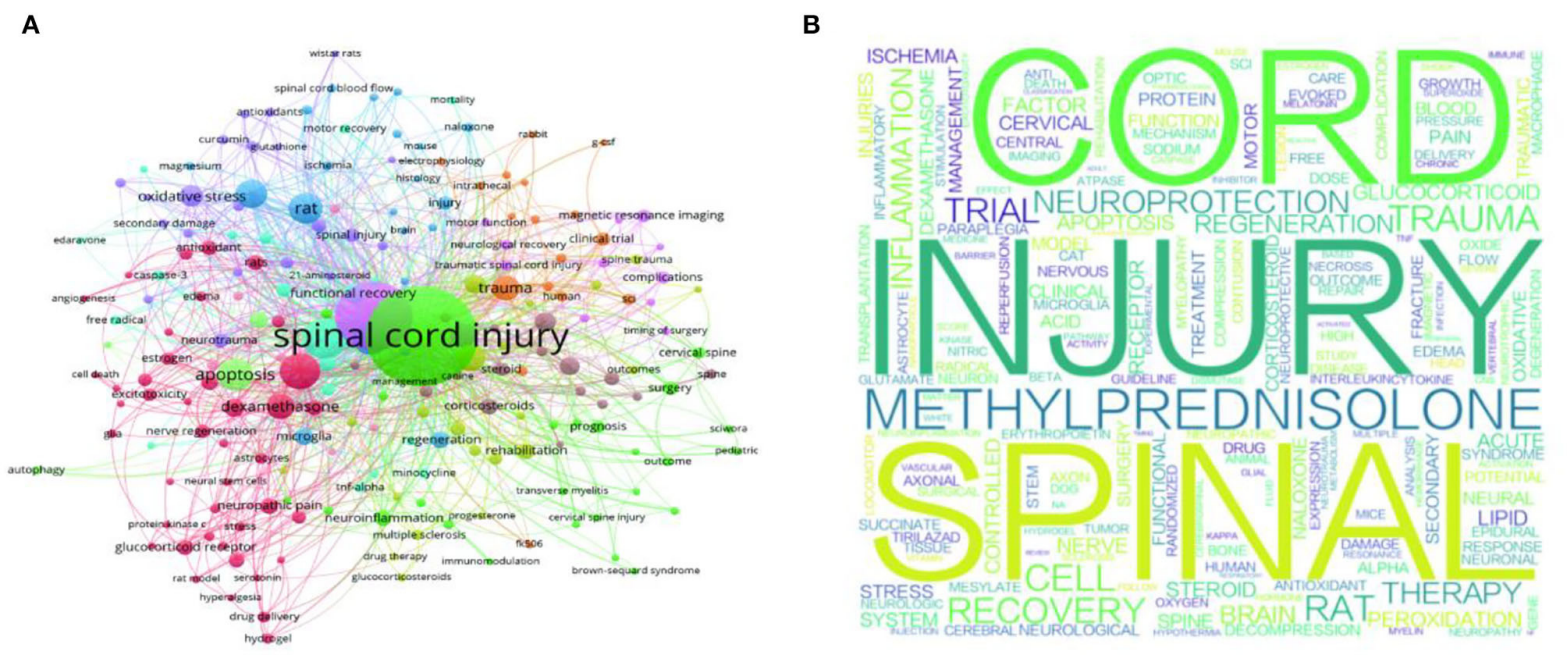
Mapping on analysis of keywords in spinal cord injury and glucocorticoid publications. **(A)** Co-occurrence of keywords. **(B)** Word cloud. “spinal cord injury,” “MP,” “neuroprotection,” and “inflammation” occurred most commonly.

The keywords in published papers that appeared more than five times in the WOS core database were selected for the final analysis. Among 3,087 keywords, 209 met the threshold. The most apparent keywords were SCI (total link strength of 1,341) and MP (total link strength of 762), both of which were strongly linked to neuroprotection and inflammation. For the comparison of SCI, apoptosis and trial were the additional keywords used, which had a link strength of >200 (Figure 2A). Similarly, we created a word cloud that reflected a visual representation of keywords that appeared more than ten times. Nevertheless, the analysis indicated that SCI and MP were the most frequent keywords, followed by trauma, recovery, and inflammation (Figure 2B).

### Bibliometric Analysis of Publications, Attribution, and Citations From 1982 to 2022

The top 20 most cited articles in terms of SCI and glucocorticoid are presented in Table 1. Most were clinical trial studies and reviews. Concurrently, the rest was experimental research articles that mainly focused on neuroprotection, inflammation, and animal model studies. The mean citation count for the top 20 most-cited articles was 682 (range: 358–1828). All papers were published between 1990 and 2017, and the most journals were the Journal of Neuroscience, with four papers.

**Table 1:** Top 20 articles on Spinal cord injury (SCI) and glucocorticoid in Web of Science from 1982 to 2022.

**Titles**	**Journals**	**Publication years**	**Total citations**	**Average per Year**
A randomized, controlled trial of methylprednisolone or naloxone in the treatment of acute spinal cord injury. Results of the Second National Acute Spinal Cord Injury Study (10).	The New England Journal of Medicine	1990	1828	55.39
Graded histological and locomotor outcomes after spinal cord contusion using the NYU weight-drop device versus transection (13).	Experimental Neurology	1996	1139	42.19
Reactive astrocytes protect tissue and preserve function after spinal cord injury (14).	Journal of Neuroscience	2004	1084	57.05
Administration of methylprednisolone for 24 or 48 h or tirilazad mesylate for 48 h in the treatment of acute spinal cord injury—Results of the Third National Acute Spinal Cord Injury Randomized Controlled Trial (15).	JAMA	1997	952	36.62
Apoptosis and delayed degeneration after spinal cord injury in rats and monkeys. (16).	Nature Medicine	1997	912	35.08
Epidemiology, demographics, and pathophysiology of acute spinal cord injury (17)	Spine	2001	827	37.59
CNS injury, glial scars, and inflammation: Inhibitory extracellular matrices and regeneration failure (18)	Experimental Neurology	2008	713	47.53
Microglia activated by IL-4 or IFN-gamma differentially induce neurogenesis and oligodendrogenesis from adult stem/progenitor cells (19)	Molecular and Cellular Neuroscience	2006	634	37.29
Effect of intravenous corticosteroids on death within 14 days in 10008 adults with clinically significant head injury (MRC CRASH trial): randomized placebo-controlled trial (20)	Lancet	2004	625	32.89
Guidelines for the conduct of clinical trials for spinal cord injury as developed by the ICCP panel: spontaneous recovery after spinal cord injury and statistical power needed for therapeutic clinical trials (21)	Spinal Cord	2007	555	34.69
Early vs. Delayed Decompression for Traumatic Cervical Spinal Cord Injury: Results of the Surgical Timing in Acute Spinal Cord Injury Study (STASCIS)(22)	Plos One	2012	544	49.45
Spinal-cord injury (23)	Lancet	2002	537	25.57
Methylprednisolone or naloxone treatment after acute spinal cord injury: 1-year follow-up data. Results of the second National Acute Spinal Cord Injury Study (24)	Journal of Neurosurgery	1992	518	16.71
Traumatic spinal cord injury (25)	Nature Reviews Disease Primers	2017	429	71.5
From basics to clinical: A comprehensive review on spinal cord injury (26).	Progress in Neurobiology	2014	417	46.33
Experimental modeling of spinal cord injury: Characterization of a force-defined injury device (27).	Journal of Neurotrauma	2003	401	20.05
Recovery of motor function after spinal-cord injury–a randomized, placebo-controlled trial with GM-1 ganglioside (28).	The New England Journal of Medicine	1991	395	12.34
Schwann cell but not olfactory ensheathing glia transplants improve hindlimb locomotor performance in the moderately contused adult rat thoracic spinal cord (29).	Journal of Neuroscience	2002	386	18.38
The neuroprotective pharmacology of methylprednisolone (30).	Journal of Neurosurgery	1992	386	12.45
MASCIS evaluation of open field locomotor scores: Effects of experience and teamwork on reliability (31).	Journal of Neurotrauma	1996	358	13.26

**Figure 3:**
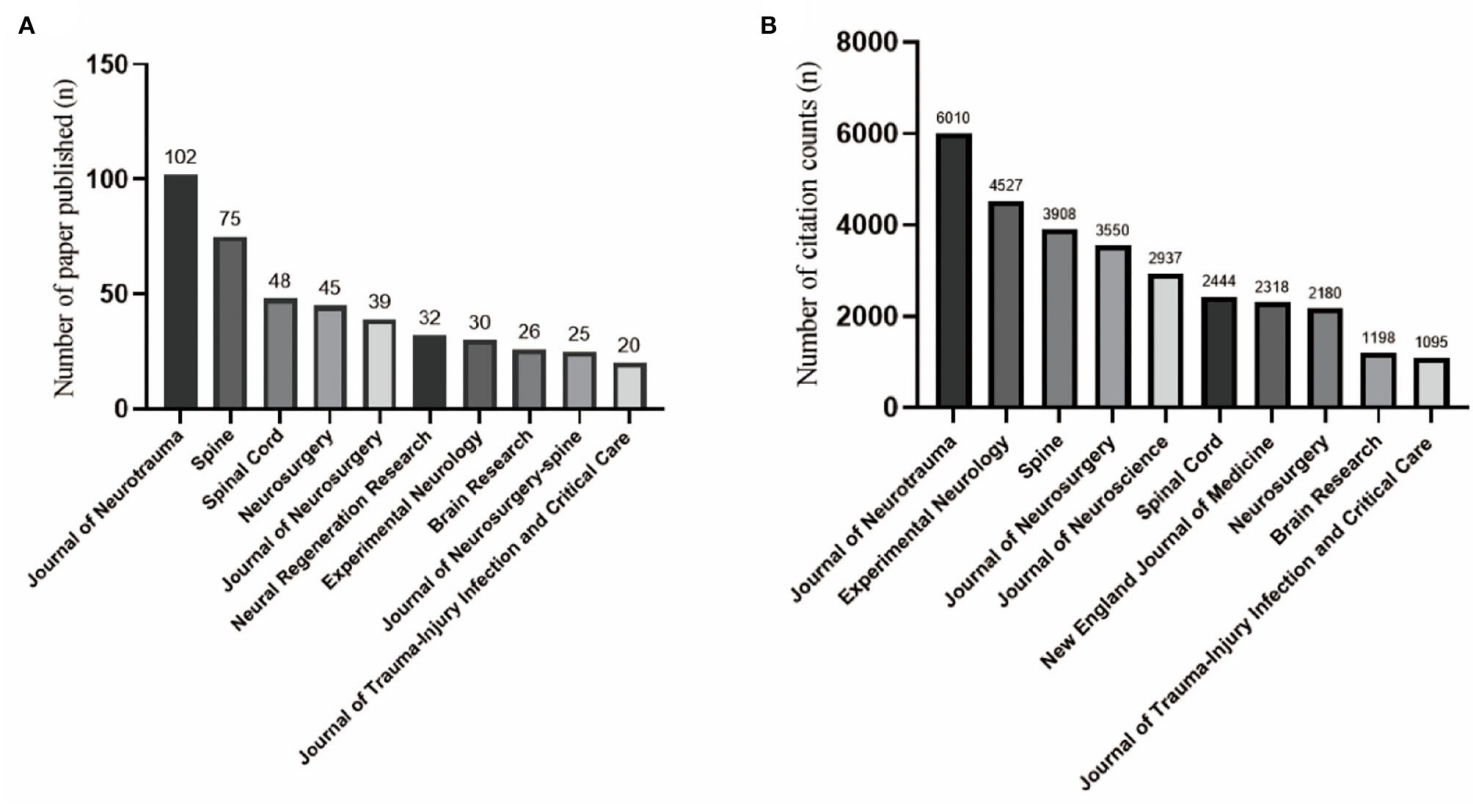
**(A)** Histogram of the top ten most-cited journals in the field of spinal cord injury and glucocorticoids, with journals of neurotrauma, spine, and spinal cord in the top three. **(B)** Top ten most published journals, with Journal of Neurotrauma having far more articles than any other nine journals, reaching 6,010.

A total of 638 journals have published papers about SCI and glucocorticoids, among which 36 published more than five relevant articles. A total of 434 relevant articles were published in the top ten active journals, accounting for 68.03% of publications in the WOS core database. The Journal of Neurotrauma was the highest-ranking journal in terms of the number of articles published, with a current impact factor (IF) of 5.269 and 102 relevant articles published from 1982 to 2022, followed by Spine with 75 publications and an IF of 3.468. The most-cited journal was the Journal of Neurotrauma which received 6,010 citations, followed by Experimental Neurology with 4,527 citations and an IF of 5.33 (Figure 3).

**Figure 4:**
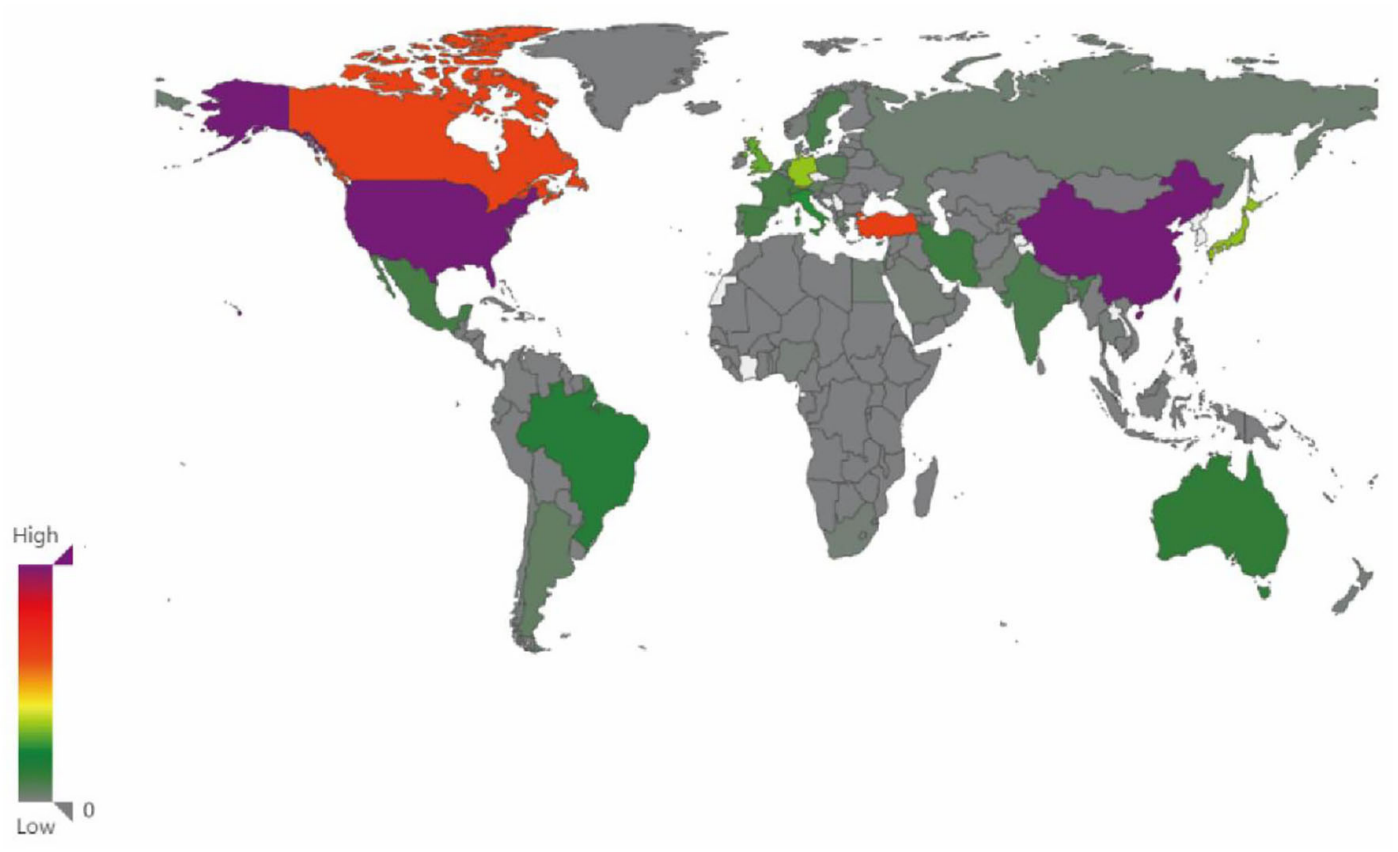
The distribution of the top productive world countries on papers. The top 5 productive countries were USA, China, Turkey, Canada, and Japan.

**Figure 5:**
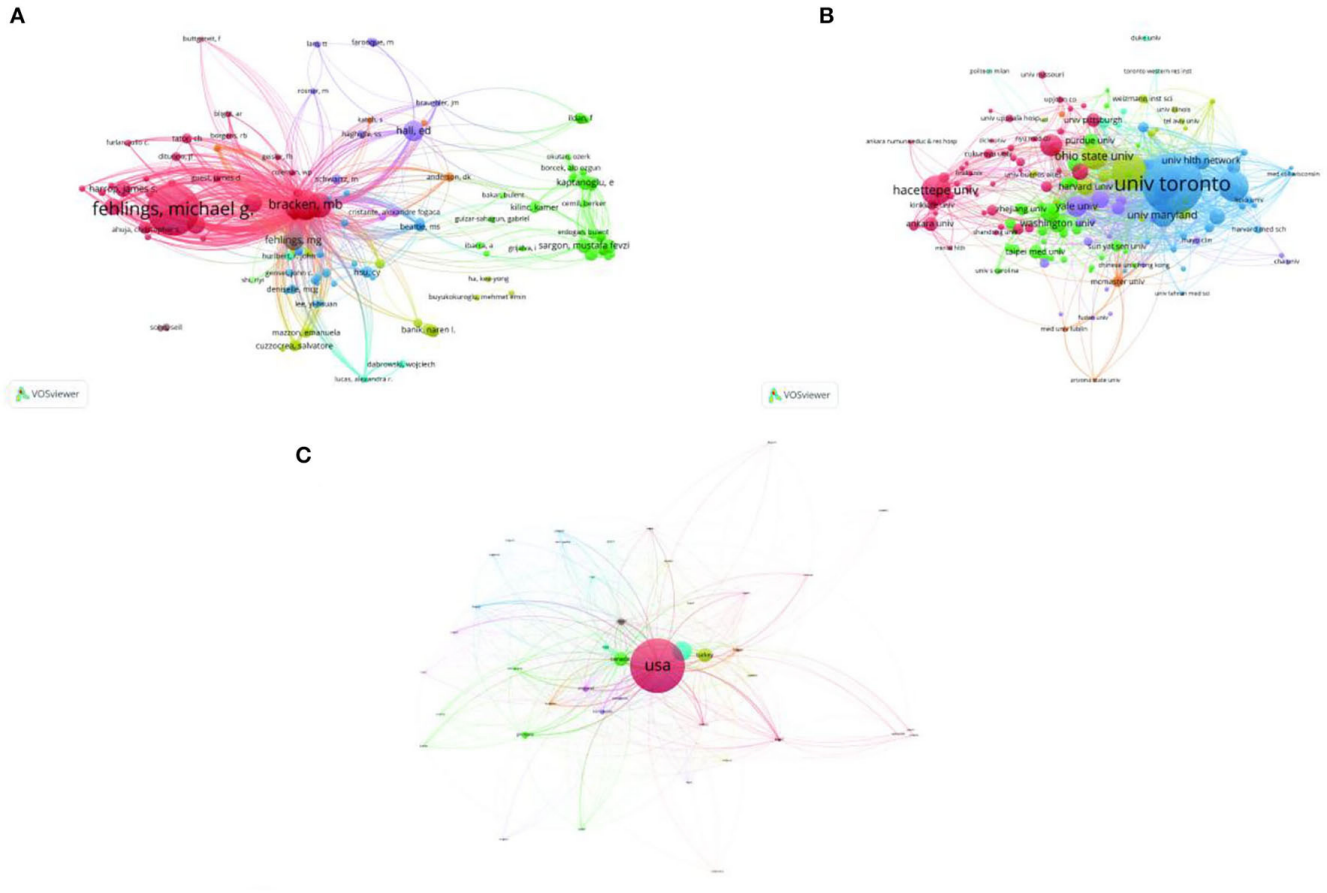
Bibliometric analysis of citations. **(A)** Mapping on cited authors shows that Michael G Fehlings of the University of Toronto is the most cited author. **(B)** Among cited institutions, the University of Toronto in the blue cluster is the most cited organization. **(C)** Mapping on cited countries, the USA is the most cited country in the field.

Based on the number of publications, the top ten countries, organizations, and authors in SCI and glucocorticoids are listed in Table 2. The results indicated that 90 articles were published by the University of Toronto and cited 7,632 times, a top-ranked-cited organization. Among 90 articles, 69 were written by Michael G Fehlings and cited 6,092 times. The 65 articles were published by the University of California with 5,287 citations. The production of different countries is represented in Figure 4 according to their distribution on the world map, with different colors used to represent the order of magnitude of output. Furthermore, 800 papers were published in the United States with total citations of 39,633 and a total link strength of 5,714, which conferred the US the top rank, followed by China with 241 published papers and 3,403 citations (Figure 5).

**Table 2:** The top ten countries, organizations, and authors of spinal cord injury and glucocorticoid publications.

**Subject**	**Number of publications**	**Count of citations**	**H-index**
**Countries/Region**			
USA	800	39633	106
China	241	3403	28
Turkey	166	3512	33
Canada	166	11011	57
Japan	85	3850	34
Germany	88	3562	28
England	68	5189	30
Italy	62	3262	27
South Korea	51	1972	22
Taiwan	38	949	20
**Organizations**			
University of Toronto	90	7632	41
University of California System	65	5287	28
University Health Network Toronto	63	5675	34
Jefferson University	52	3311	26
University of Miami	45	2471	22
Hacettepe University	41	912	19
University of British Columbia	40	3148	24
Us Department of Veterans Affairs	38	1398	18
Veterans Health Administration VHA	38	1398	18
Yale University	36	5594	21
**Authors**			
Fehlings, michael G	69	6092	36
Bracken, MB	31	5054	17
Young, W	23	5182	18
Hall, ED	21	1809	16
Sargon, Mustafa Fevzi	20	327	13
Banik, NL	19	898	16
Vaccaro, Alexander R	19	459	12
Kwon, BK	18	782	13
Kilinc, K	16	341	12
Wilson, Jefferson R	16	1434	13

**Figure 6:**
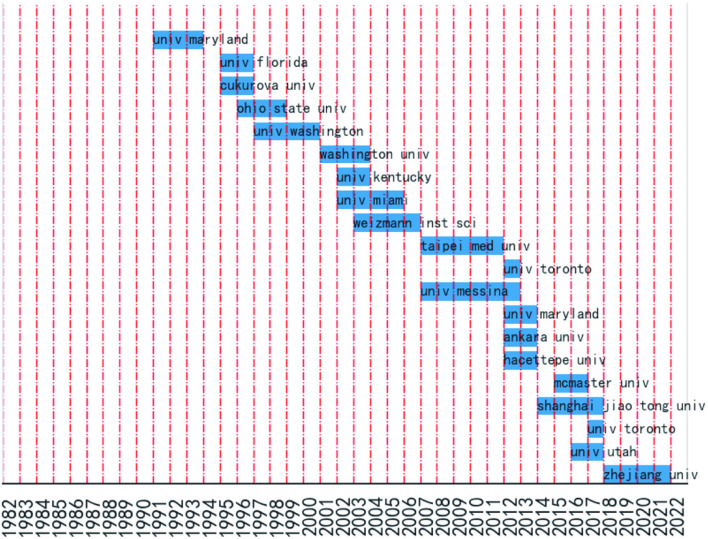
Burst detection analysis of organizations.

Burst detection analysis results of organizations are indicated in Figure 6, showing that Zhejiang University China has made more contributions and achievements in SCI and glucocorticoids research in recent years, followed by Shanghai Jiao Tong University, China.

**Figure 7:**
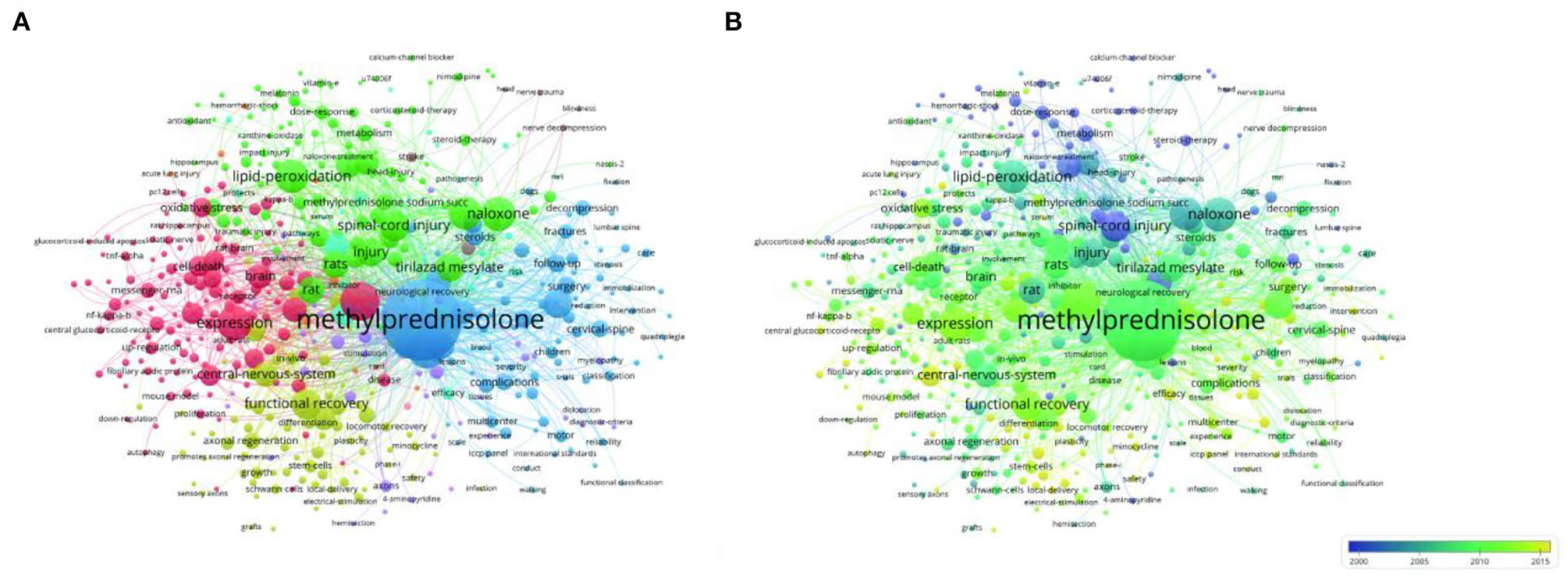
**(A)** Distribution of the themes. The yellow cluster represents studies of recovery of nerve function. The blue cluster indicates diagnosis and clinical features. The red cluster involves molecular regulatory mechanisms. **(B)** Average year map of keywords used from 1982 to 2022.

As indicated in Figure 7A, four themes of SCI and glucocorticoid studies were observed. The blue cluster involved SCI diagnosis and clinical features. The red cluster involved animal models and key molecular regulatory mechanisms. The yellow cluster involved recovery of nerve function and stem cell transplant and the green cluster involved clinical effects and complications of high-dose MP application. Concurrently, Figure 7B demonstrates the network map of trend topics according to the keywords used from 1982 to 2022, where purple to yellow indicators show publications. The highly cited literature belonged to the pre-2015 era, with research mainly focused on the molecular mechanisms of neurological recovery and the study of neural stem cells.

According to the clinical research topic of the green cluster shown in Figure 7, we used the Cochrane Handbook and Newcastle–Ottawa Scale method to screen relevant clinical research literature, included all eligible randomized, controlled trials (RCTs), and controlled observational studies that compared MPS against placebo or no treatment in adult patients with acute SCI (11, 32). As shown in Table 3, according to the results of clinical use of methylprednisolone in SCI patients, it can be seen that there are different prognosis conclusions, and the time span is from year 1990 to 2019. There were 17 of these eligible clinical studies, eight in the United States and four in Japan. There are 3 studies, including the NASCIS II study that suggests that MP can improve neurological function in SCI patients, two of which are RCT studies. However, 11 studies, including 1 RCT, concluded that it did not improve neurological function. The studies that concluded that the use of high-dose MP resulted in increased complications in SCI patients accounted for 53.3% (8/15) of all studies, including two RCTs. Among these studies, the H-index of The New England Journal of Medicine published by Bracken et al. (10) was significantly higher than that of other journals, and the H-index of journals published by other institutes was almost at the same level.

**Table 3:** Literature comparison methylprednisolone applied to clinical outcomes of patients with SCI.

**References**	**Journal**	**Country**	**Publication year**	**Publication types**	**Groups size**		**outcomes**		**Total citations**	**H-index of Journal**
					**MP**	**control**	**difference in neurological function**	**difference in adverse events**		
Bracken. (10)	The New England Journal of Medicine	USA	1990	RCT	162	171	YES	NO	1828	993
Satoshi Tsutsumi et al. (33)	Spine	Japan	2006	Observational study	37	33	YES	NO	37	228
Wang et al. (34)	Pakistan Journal of Medical Sciences	China	2019	RCT	39	39	YES	NO	4	26
Sunshine et al. (35).	Anesthesia and Analgesia	USA	2017	Observational study	160	151	NO	NO	12	187
Evaniew et al. (32).	Journal of Neurotrauma	Canada	2015	Observational study	44	44	NO	YES	79	132
Khan et al. (36).	Spinal Cord	USA	2014	Observational study	216	134	Not Covered	YES	16	97
Ito et al. (37).	Spine	Japan	2009	Observational study	38	41	NO	YES	66	228
Suberviola et al. (38)	Injury	Spain	2008	Observational study	59	23	NO	YES	70	109
Pollard et al. (39)	Spine	USA	2003	Observational study	104	200	NO	Not Covered	116	228
Heary et al. (40)	Neurosurgery	USA	1997	Observational study	31	193	NO	NO	69	183
Gerndt et al. (41)	The journal of Trauma	USA	1997	Observational study	93	47	Not Covered	YES	110	168
Levy et al. (42)	Neurosurgery	USA	1996	Observational study	55	181	NO	NO	73	183
Prendergast et al. (43)	The journal of Trauma	USA	1994	Observational study	29	25	NO	Not Covered	34	168
Matsumoto et al. (44)	Spine	Japan	2001	RCT	23	23	Not Covered	YES	131	228
Pointillart et al. (45)	Spinal Cord	France	2000	RCT	27	25	NO	YES	189	97
Chikuda et al. (46)	Emergency Medicine Journal	Japan	2014	Observational study	824	800	NO	YES	64	71
Ilik et al. (47)	The Turkish Journal of Trauma and Emergency Surgery	Turkey	2019	Observational study	95	87	NO	YES	29	20

*MP, methylprednisolone; RCT, randomized controlled trial*.

## Discussion

Spinal cord injury remains a thorny public health problem globally. Various academic institutions join hands to find an appropriate treatment protocol for SCI. The medical journals with high impact, namely the New England Journal of Medicine and JAMA, have already reported the therapeutic effect of MP in SCI treatment in the early 80s. This study analyzed 1,848 publications about SCI and glucocorticoids indexed in WOS core database. The published literature included three aspects: clinical characteristics, animal experimentation, and molecular mechanisms. The most frequent keyword “spinal cord injury” is strongly linked to neuroprotection and inflammation.

Bibliometric analysis of publications, attribution, and citations gives us a holistic view of the development of the field. The Journal of Neurotrauma had published more than 102 relevant articles, far more than the 75 articles in the second place in Spine journal. Experimental Neurology had the highest average number of citations per article at 150. Six of the top ten impact institutions are located in the USA, with 800 publications in the field, more than the other nine countries combined. The United States has a high H-index of 106, indicating that it is the leader in this field. China has the second-highest number of publications, with 241, but it lags in citations and H-index. Canada's 166, and Canada's H-index of 57, is a close second to that of the USA. Although the USA has contributed to this important field, the most-cited article came from the University of Toronto, Canada. In terms of several publications, citations, and H-index, the top three authors were Fehlings, Michael G, Bracken, MB, and Young, W; all of them were from Canadian and US university institutions. This laid the foundation for the protocol for using MP, which also led to a rapid rise in SCI articles related to MP from 1990 onward, reaching 70 articles per year. In recent years, Zhejiang University and Shanghai Jiao Tong University in China have been outstanding research contributors in this field. Since 2011, Zhejiang University has published 14 articles with an average citation frequency of 12.29 and an H-index of 8. Its publications focused on animal experiments on different drugs for SCI and the application of materials science in SCI; Shanghai Jiao Tong University published 12 articles with an average citation frequency of 20.67 and an H-index of 7. Its publications include stem cell transplantation, molecular mechanisms of SCI, the application of materials science in SCI, and clinical studies on SCI. Other leading Chinese institutions in this field include Sun Yat-sen University and Nanjing University, but it can be found that Chinese institutions have a late start in this field and their academic results remain far from those of the USA and Canada.

The protocol for the clinical application of MP stems from the completion of three large randomized clinical trials, the National Acute Spinal Cord Injury Study (NASCIS). The second of these NASCIS studies had the greatest impact on using MP in clinical practice (10, 15, 48), which were the most highly cited papers in SCI treatment utilizing MP. This result led to the widespread use of MP 24-h treatment protocol (30 mg/kg bolus injection followed by a 5.4 mg/kg/h infusion over 23 h) for SCI patients who arrived at the hospital within 8 h of injury (10, 24). The NASCIS III study from 16 acute spinal cord injury centers in North America in 1997 even suggested that using MP was initiated in a 3–8 h time frame after SCI injury and that MP treatment group had better neurological recovery performance with continued use for 48 h (15).

However, using high-dose MP through intravenous administration in acute SCI has become controversial due to the risk of potential side effects and small positive effect sizes. It was thus reasonable to speculate that most side effects of high-dose MP therapy are tightly linked to the high systemic dosage of MP, which resulted in further health complications especially greater risks of gastrointestinal tract bleeding (49), aseptic necrosis of femoral head (50), and destruction of neurons and glia (51). Increasingly, voices have begun to question the reliability of the results of the second NASCIS survey study, with animal models and *in vitro* neuronal research indicating that high MP doses do not significantly promote neuronal cell proliferation (52) and could not result in significant improvements in neurological recovery in SCI model animals (53).

The efficacy of the NASCIS-II regimen was also questioned by the results of several clinical studies, including RCT conducted by T Matsumoto, which concluded that elderly patients with cervical spinal cord injury were more likely to experience pulmonary side effects after high-dose MPSS treatment (44), in addition to RCT conducted by V Pointillart, which did not observe any particular signs of neurological improvement with MP (45). Many retrospective studies have concluded that using MP significantly increases associated complications and does not improve exercise scores in SCI patients (32, 33, 35–37, 39–43). However, the authors of this manuscript found that most of these questionable studies were retrospective studies with relatively weak-quality evidence which were assessed using the Newcastle–Ottawa Scale, and small sample sizes and that confounding factors regarding age, severity of SCI, timely and comprehensive assessment of motor and sensory recovery, and timing of surgical interventions that may affect the neurological prognosis of SCI patients could not be controlled. Furthermore, most studies did not specify care details to prevent complications. All factors that were not mentioned in most retrospective studies and could not be controlled for confounding included the use or non-use of neurotrophic drugs and their duration, strict adherence to NASCIS-II protocol, using urinary catheterization, ventilators, proton pump inhibitors, and whether the patient underwent regular rehabilitation exercises during the recovery phase. The small size of each study, combined with the presence of more confounding factors, may also be a cause of biased information and inconsistent conclusions. These clinical studies had been conducted in various parts of the world, such as North America, Asia and Europe, and it is possible that ethnic differences may affect the results to some extent. Interestingly, the authors of this paper also checked for conflicts of interest in these clinical studies and found that the methylprednisolone used in a study was provided by the Upjohn Corporation (10). In a separate study, Pharmacia and Upjohn Inc funded additional tests and monitored data quality at participating centers (15).

Despite this, there was a change in the views of frontline surgeons on the need for high-dose use of MP therapy when treating SCIs (54, 55). By balancing the available perspectives and evidence, the 2013 AANS/CNS SCI guidelines discourage MP use in SCI and downgrade it from a Class I evidence level to Class III (56). However, in the absence of new robust evidence in this field, Michael G Fehlings published AOSpine guidelines in the Global Spine Journal in 2017, which still recommends offering patients a 24-h infusion of MP as a treatment option within 8 h of acute SCI (57). The 2019 guidelines from China on spinal cord injury suggest that high-dose MP therapy is no longer routinely used for acute SCI, but remains an optional treatment in some cases (58). However, French recommendations for the management of patients with spinal cord injury are that steroids are not recommended for early use to improve neurological prognosis (59). As a result, spinal or neurosurgical surgeons worldwide continue to find it tricky to use MP in SCI patients. Moreover, most developing countries often feel helpless in responding to the 8-h MP treatment protocol proposed by the US Acute Spinal Cord Injury Center. Due to the relative paucity of medical resources and the inadequacy of the medical system, developing countries are not as responsive to acute patients as developed countries, and most SCI patients often arrive at the hospital more than 8 h later. The choice of their medication regimen is also another major challenge.

Therefore, further prospective randomized cohort studies are required to confirm and update the current conclusions regarding the weak quality of the currently questioned studies to increase the sample size and better control for potential confounding factors such as age, the severity of injury, and duration of intervention.

## Conclusions

With an ever-increasing number of publications about using MP in SCI, it is important to assess the quality of such many research papers and gain valuable information. Although scientific and medical research plays a vital role in understanding SCIs, this study demonstrated the need for a more rigorous prospective clinical trial design to validate the role of MP in SCI and explore more rational use protocols.

## Data Availability Statement

The original contributions presented in the study are included in the article/supplementary material, further inquiries can be directed to the corresponding author.

## Author Contributions

YZ and A-AL conceived and designed the study, conducted the data collection, and wrote the manuscript. S-NX, N-SZ, W-LT, and S-JW performed the analysis and generated the figures and tables. J-ML and Z-LL critically reviewed the manuscript. All authors have read and approved the manuscript.

## Funding

This work was supported by the Double Thousand Plan Talent Project of Jiangxi Province and Department of Science and Technology Program of Jiangxi Province, China (No. 20203BBG73045).

## Conflict of Interest

The authors declare that the research was conducted in the absence of any commercial or financial relationships that could be construed as a potential conflict of interest.

## Publisher's Note

All claims expressed in this article are solely those of the authors and do not necessarily represent those of their affiliated organizations, or those of the publisher, the editors and the reviewers. Any product that may be evaluated in this article, or claim that may be made by its manufacturer, is not guaranteed or endorsed by the publisher.
